# High-intensity interval training induces lactylation of fatty acid synthase to inhibit lipid synthesis

**DOI:** 10.1186/s12915-023-01698-9

**Published:** 2023-09-19

**Authors:** Xuefei Chen, Wenhua Huang, Jingbo Zhang, Yanjun Li, Zheng Xing, Lanlan Guo, Hongfeng Jiang, Jing Zhang

**Affiliations:** 1 College of P.E. and Sports, Beijing Normal University, Beijing, 100875 China; 2 School of Physical Education Institute (Main Campus), Zhengzhou University, Zhengzhou, China; 3 Experimental Research Center, Beijing Institute of Heart, Lung and Blood Vessel Diseases, Beijing Anzhen Hospital, Capital Medical University, Beijing, China

**Keywords:** High-intensity interval training, Lactylation, Fatty acid synthase, Fat loss

## Abstract

**Background:**

The aim of study was to observe the effect of increased lactate levels during high-intensity interval training (HIIT) on protein lactylation, identify the target protein, and investigate the regulatory effect of lactylation on the function of the protein.

**Methods:**

C57B/L6 mice were divided into 3 groups: the control group, HIIT group, and dichloroacetate injection + HIIT group (DCA + HIIT). The HIIT and DCA + HIIT groups underwent 8 weeks of HIIT treatment, and the DCA + HIIT group was injected DCA before HIIT treatment. The expression of lipid metabolism-related genes was determined. Protein lactylation in subcutaneous adipose tissue was identified and analyzed using 4D label-free lactylation quantitative proteomics and bioinformatics analyses. The fatty acid synthase (FASN) lactylation and activity was determined.

**Results:**

HIIT had a significant effect on fat loss; this effect was weakened when lactate production was inhibited. HIIT significantly upregulated the protein lactylation while lactate inhibition downregulated in iWAT. FASN had the most modification sites. Lactate treatment increased FASN lactylation levels, inhibited FASN activity, and reduced palmitate and triglyceride synthesis in 3T3-L1 cells.

**Conclusions:**

This investigation revealed that lactate produced by HIIT increased protein pan-lactylation levels in iWAT. FASN lactylation inhibited de novo lipogenesis, which may be an important mechanism in HIIT-induced fat loss.

**Supplementary Information:**

The online version contains supplementary material available at 10.1186/s12915-023-01698-9.

## Highlights

1. Lactate is involved in the fat-reducing effect of HIIT.

2. Lactate produced by HIIT induces protein lactylation in subcutaneous adipose tissue of mice.

3. Lactate induces FASN lactylation, which inhibits its activity and function.

**Graphical Abstract:**

**Supplementary Information:**

The online version contains supplementary material available at 10.1186/s12915-023-01698-9.

## Graphical abstract

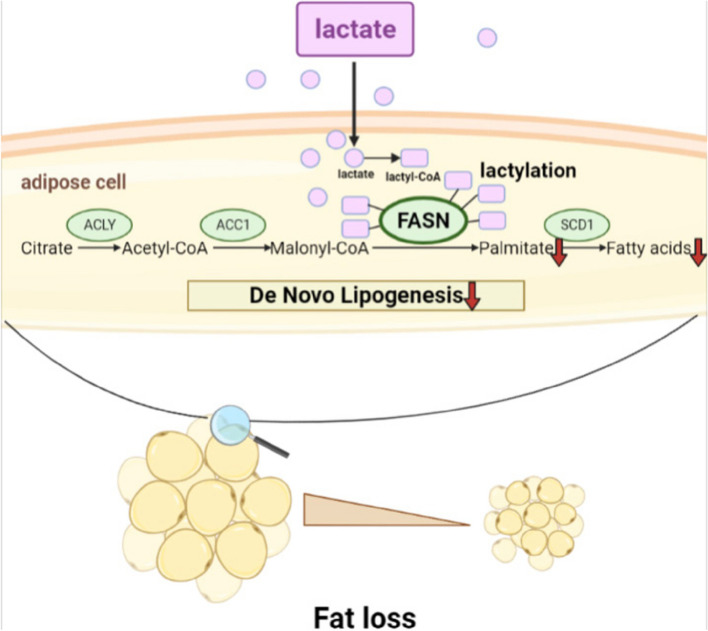

## Background

HIIT is a training modality that consists of repeated, brief bouts of exercise at high intensity interspersed with periods of rest or low-intensity exercise [1]. In recent years, it has been reported that HIIT can have a strong fat-reducing effect and has a prominent effect on visceral adipose tissue, subcutaneous adipose tissue, and total adipose tissue [2]. “Fat burning” has become a hallmark effect of HIIT, but the key molecules involved in regulation and its mechanism have not been elucidated [3]. Lactate is the iconic product of HIIT and is formed by the combination of lactate anions and hydrogen ions. In the pH environment of the body, almost all lactate exists in a free state [4]. Lactate produced by skeletal muscle during HIIT is absorbed and utilized by various tissues and cells through the cell–cell lactate shuttle and intracellular lactate shuttle monocarboxylate transporter-1 (MCT-1) [5]. In addition, Cai et al. reported that lactate can be used as a signaling molecule and is a specific ligand of Gi-protein-coupled receptor 81 (GPR81) [6]. Via GPR81 binding, lactate exerts various biological effects. Lactate can also induce a lipid metabolic reprogramming which results into the formation and mobilization of lipid droplets [7]. However, the role of lactate in fat–mass loss after HIIT stimulation has not been reported.

Recently, a study published in *Nature* reported that the lactate produced by glycolysis acts as a precursor that stimulates histone lactylation [8]. HIIT could repeatedly expose the body to a high lactate environment; will it induce an increase in protein lactylation? What kind of biological effect will it have? None of the answers to these questions have been reported. In the present study, we established a mice HIIT model to observe the role of lactate in HIIT-promoting fat loss and assessed its effect on protein lactylation in tissues. Meanwhile, target proteins for lactylation were identified via 4D label-free lactylation quantitative proteomics to clarify the effect of lactylation on the function of target proteins and to explore the effect of HIIT-induced enhancement of lactylation on lipid metabolism.

## Results

### Lactate is involved in the fat loss induced by HIIT

**Fig. 1:**
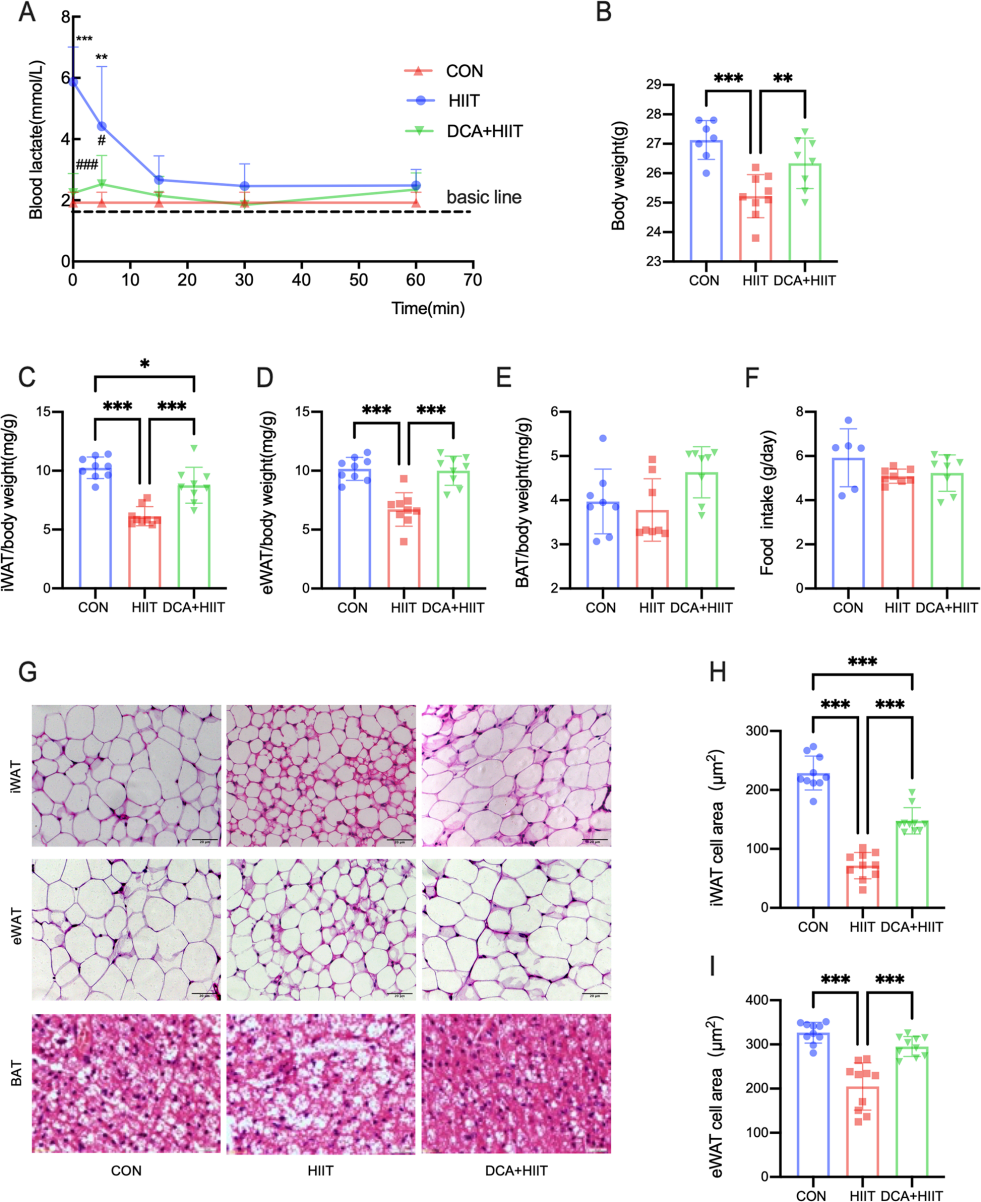
HIIT stimulates lactate secretion to induce fat loss. **A** The level of blood lactate before and after exercise for 0, 5, 15, 30, and 60 min. Data are shown as mean ± SD, *n* = 7–10. **B** Body weight of mice under different intervention methods. **C–E** The ratio of iWAT, eWAT, and BAT weight to body weight in mice. **F** Food intake of CON, HIIT, and DCA + HIIT mice was monitored. **G** H&E staining results for iWAT, eWAT, and BAT (40X). **H**, **I** Quantification of the average cross-sectional area of iWAT and eWAT. Data are shown as mean ± SD, *n* = 10, *p < 0.05; **p < 0.01; ***p < 0.001. HIIT high-intensity interval training, DCA + HIIT dichloroacetate injection + high-intensity interval training, iWAT subcutaneous adipose tissue, eWAT visceral adipose tissue, BAT brown adipose tissue

After exercise, the blood lactate level in HIIT group was significantly upregulated and returned to normal levels in 1 h, while the blood lactate level in DCA + HIT group showed no obvious change (Fig. 1A). We observed lower body weight, iWAT/body weight, and eWAT/body weight ratios in HIIT mice while BAT/body weight had no significant change (Fig. 1B–E). The results show that there were no significant differences in the amounts of food intake among the three groups (Fig. 1F). The size of iWAT and eWAT cells was smaller in HIIT (Fig. 1G–I). To test the function of lactate, DCA was used to inhibit the product of lactate and injected intraperitoneally 10 min before HIIT. Compared with HIIT mice, the decrease of body weight, iWAT/body weight ratio, and eWAT/body weight ratio in DCA + HIIT group were inhibited (Fig. 1B–D). The decreases of iWAT and eWAT cells was prevented in DCA + HIIT (Fig. 1G–I). In addition, HIIT reduced the relative amounts of C14:0, C14:1, C16:0, C16:1, C20:0, and C22:0 fatty acids in plasma, and DCA treatment inhibited the decrease in C14:0 fatty acids (Fig. S1A). We also evaluated the expression of genes related to lipid metabolism. Cluster of differentiation 36 (CD36) expression in iWAT was upregulated in HIIT mice and downregulated in DCA + HIIT mice (Fig. S1B), but the expression of other lipogenesis-related genes was not significantly altered (Fig. S1C-F). We then measured the mRNA expression levels of key enzymes involved in fatty acid synthesis. ATP citrate lyase (ACLY) expression in iWAT was upregulated during HIIT, but the other enzymes showed no significant change (Fig. S1G-J). These results suggest that HIIT has a significant effect on fat loss and lactate might be an important factor.

### HIIT promotes protein lactylation in iWAT

**Fig. 2:**
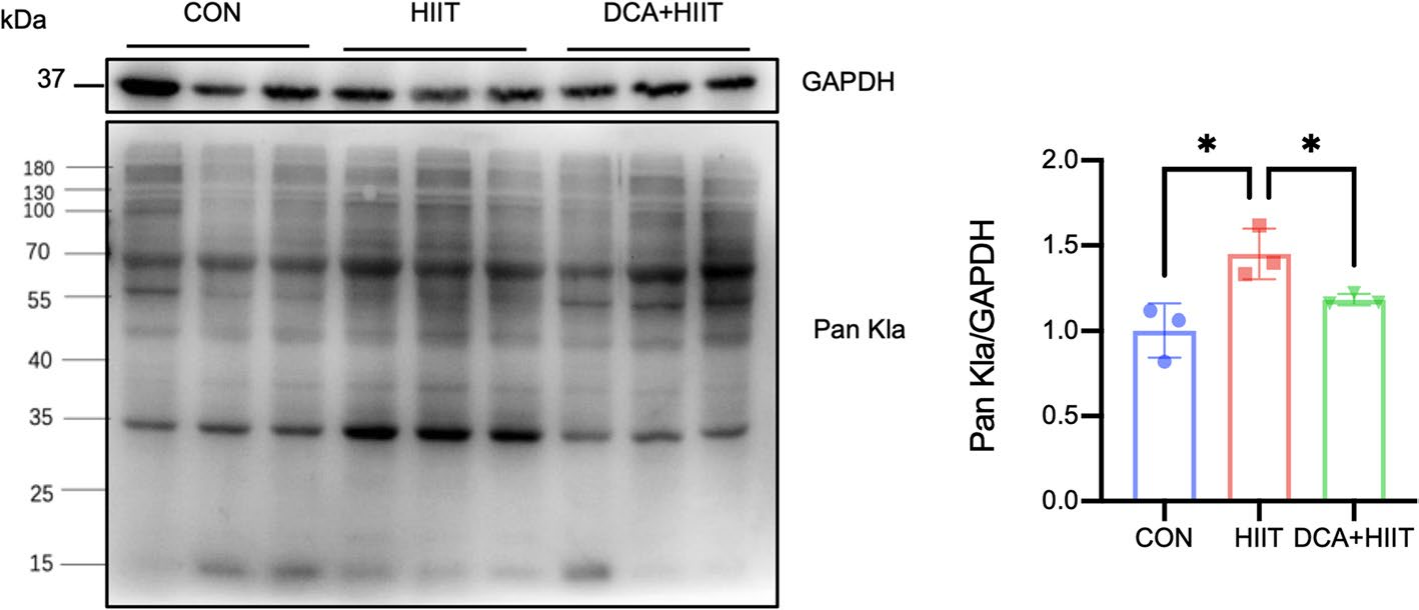
Protein lactylation in iWAT is induced by HIIT. The protein expression level of Pan Kla in iWAT was measured by western blotting under different interventions. *n* = 3. HIIT high-intensity interval training, DCA + HIIT dichloroacetate injection + high-intensity interval training, iWAT subcutaneous adipose tissue, GAPDH glyceraldehyde-3-phosphate dehydrogenase, Pan Kla pan lysine lactylation

As an important product of glycolysis, lactate induces protein lactylation in a tissue-specific manner [9] and participates in a variety of biological processes. However, whether HIIT promotes lactylation has not been reported. We next investigated the protein lysine lactylation status in different tissues from CON, HIIT, and DCA + HIIT mice. Western blot analysis for lactyllysine demonstrated that the level of lactylation increased in iWAT (Fig. 2), liver (Fig. S2B), and heart (Fig. S2C) in HIIT mice and increased most dramatically in iWAT. Compared with HIIT group, the levels of lactylation were decreased in iWAT (Fig. 2A), liver (Fig. S2B), and heart (Fig. S2C) in DCA + HIIT group.

### Quantitative proteomic analysis of lysine lactylation in mice iWAT

**Fig. 3:**
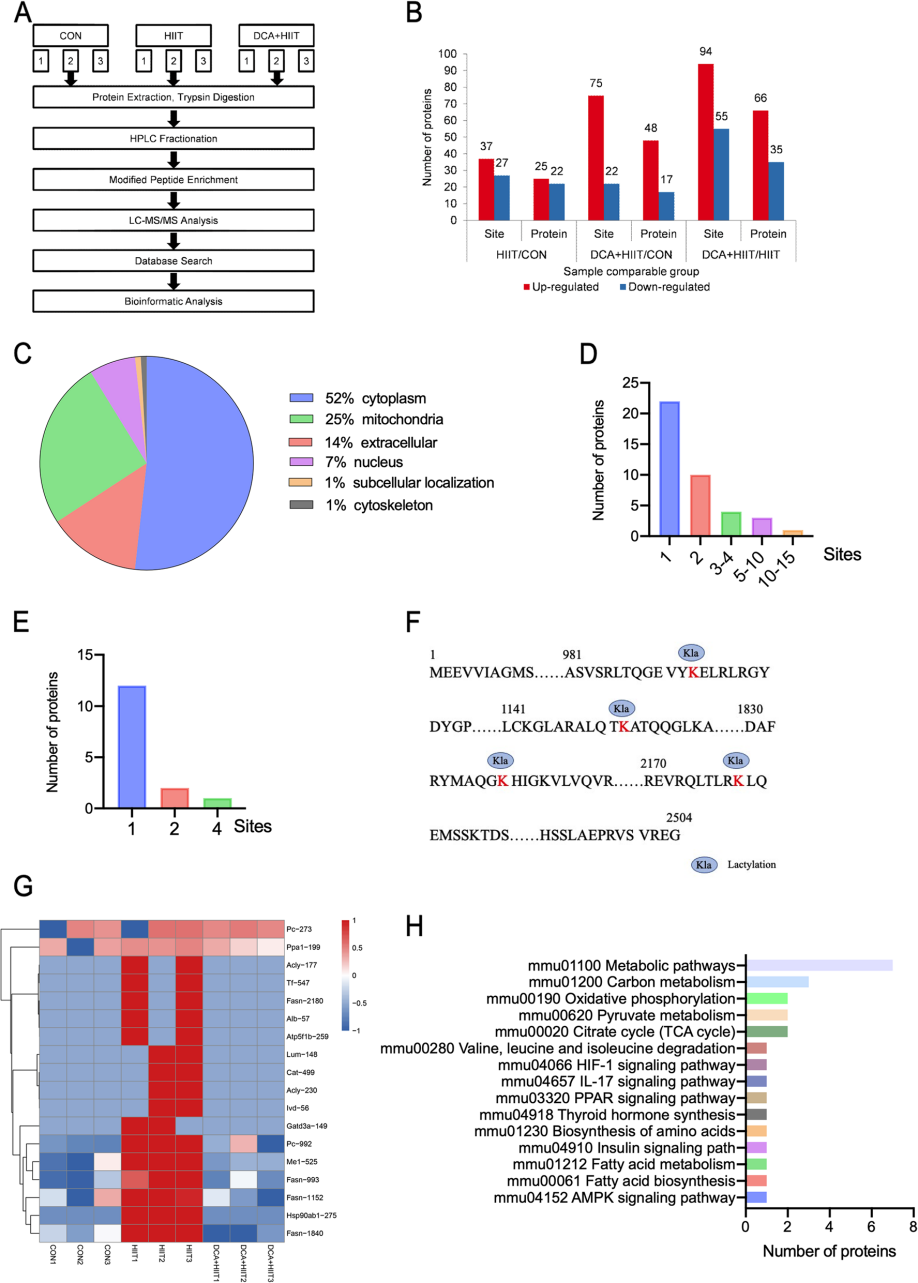
Detection and profile of lysine lactylation in mice iWAT. **A** Schematic representation of the experimental workflow for 4D label-free lactylation quantitative proteomics in CON, HIIT, and DCA + HIIT mice. **B** Distribution of the number of differentially lactylated lysine sites and proteins. **C** Pie chart of the predicted subcellular localization of lysine lactylated proteins. **D** Distribution of the number of lysine lactylation sites per protein that were significantly upregulated (fold change > 1.3, *p* < 0.05) and downregulated (fold change < 0.7, *p* < 0.05) among groups. Distribution of the number (**E**), hierarchical clustering and heatmap analysis (**G**), and KEGG pathway analysis (**H**) of lysine lactylation sites per protein that were upregulated in HIIT mice and downregulated in DCA + HIIT mice. **F** Lysine lactylation sites of FASN; *n* = 3. KEGG Kyoto Encyclopedia of Genes and Genomes, HIIT high-intensity interval training, DCA + HIIT dichloroacetate injection + high-intensity interval training

We next investigated the target proteins that are lactylated and regulated by lactate levels in mice iWAT. We used 4D label-free lactylation quantitative proteomics followed by enrichment of the lactylated peptides for identification via UPLC (Fig. 3A). SDS–PAGE and RSD analyses were conducted across all treatment groups to examine the reproducibility of our proteomic analysis (Fig. S3A, B). We identified 602 quantifiable sites and 217 quantifiable proteins (Fig. S3C).

Then, we quantitatively analyzed the changes in protein lactylation in three groups. Modification sites in both groups with *p* < 0.05 were considered significantly different modifications, and the standard for up- or down-regulated modification sites was set as a fold change > 1.3 or < 0.7, respectively. Based on the above data and standards, 37 modification sites in 25 proteins were upregulated and 27 modification sites in 22 proteins were downregulated in HIIT group vs. control group. Meanwhile, 75 modification sites in 48 proteins were upregulated and 22 modification sites in 17 proteins were downregulated in DCA + HIIT group vs. HIIT group (Fig. 3B). We examined their predicted subcellular compartments: most localized to the cytoplasm (52%), followed by mitochondria (25%), extracellular (14%), nucleus (7%), cytoskeleton (1%), and subcellular localization (1%) (Fig. 3C). Heatmap analysis and KEGG enrichment analysis demonstrated that Kla proteins were enriched in multiple primary metabolic pathways (Fig. S3D, E), such as glycolysis/gluconeogenesis, pyruvate metabolism, and the citrate cycle (TCA cycle). In contrast, fatty acid degradation and fatty acid biosynthesis were also significantly enriched in the KEGG pathway analysis, suggesting a potential role of protein Kla in the regulation of fatty acid biosynthesis and degradation.

Of these Kla proteins, 32 and 4 have 1–2 and 3–4 Kla sites, respectively, while the remaining 4 have ≥ 5 Kla sites (Fig. 3D). Across all groups, 13 proteins and 18 lactylation sites were significantly upregulated (fold change > 1.3, *p* < 0.05) in HIIT mice and downregulated (fold change < 0.7, *p* < 0.05) in DCA + HIIT group (Fig. 3E). FASN was identified to have the most lactylation sites (at 4 Lys residues) (Fig. 3F). The upregulated Kla proteins included FASN, ATP-citrate synthase, and pyruvate carboxylase (Fig. 3G). KEGG enrichment analysis demonstrated that Kla proteins were enriched in metabolic pathways and fatty acid biosynthesis (Fig. 3H). These results suggest that FASN lactylation may have a considerable impact on functionality.

### Lactate induces FASN lactylation in 3T3-L1 cells

**Fig. 4:**
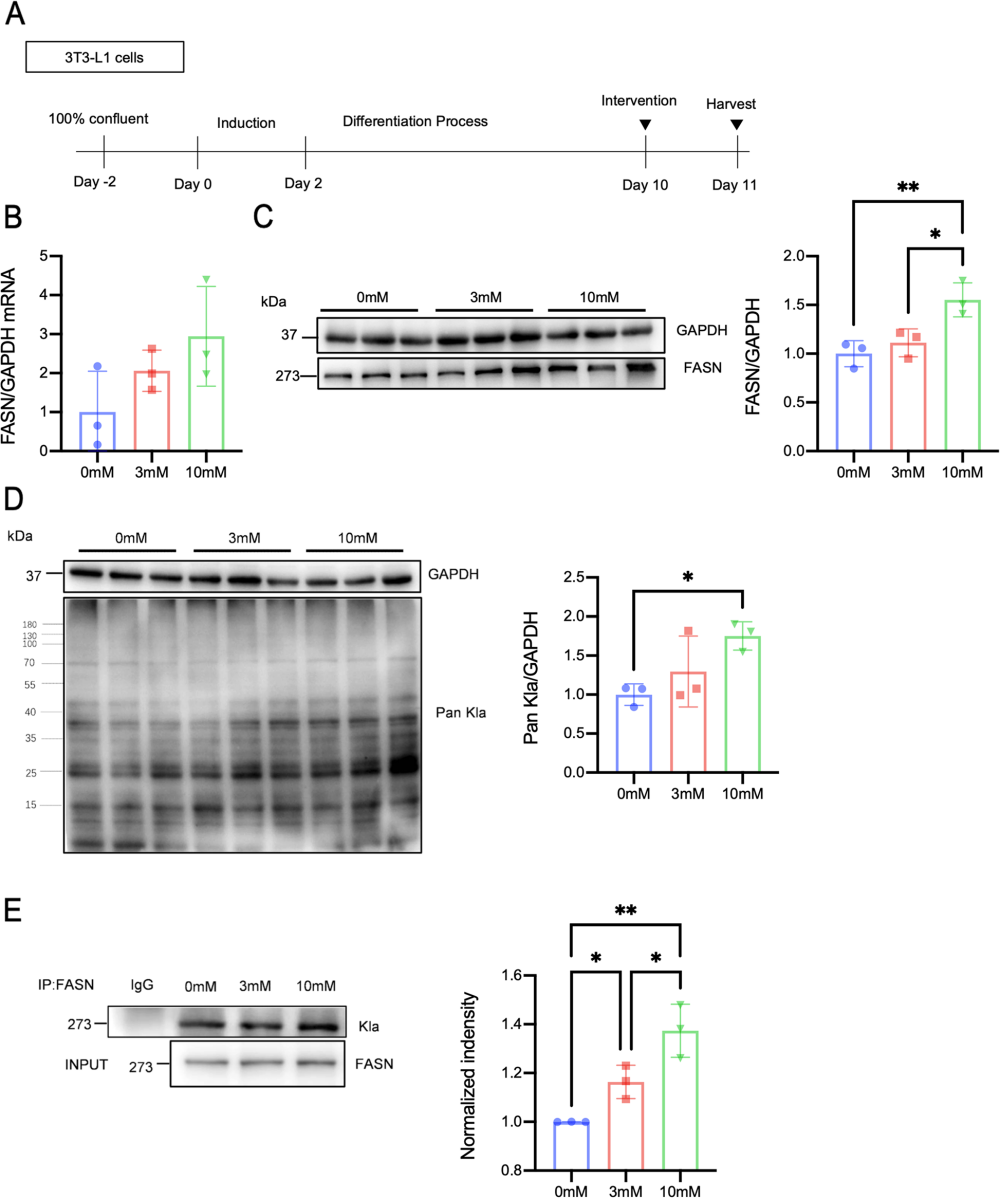
Lactate induces FASN expression and lactylation in 3T3-L1 cells. **A** 3T3-L1 preadipocytes were differentiated into mature adipocytes over a 10-day period. Then, the cells were treated with 0 mM, 3 mM, or 10 mM lactate for 24 h. **B**, **C** FASN mRNA and protein expression levels were detected in 3T3-L1 cells. **D** Pan Kla levels after 0 mM, 3 mM, and 10 mM lactate treatment were detected in 3T3-L1 cells via western blotting. **E** FASN was immunoprecipitated from 3T3-L1 cells using a FASN-specific antibody. Lactate treatment increased FASN lactylation. Data are shown as mean ± SD, *n* = 3–4; *p < 0.05, **p < 0.05. HIIT high-intensity interval training, DCA + HIIT dichloroacetate injection + high-intensity interval training, FASN fatty acid synthase, GAPDH glyceraldehyde–3-phosphate dehydrogenase, *Pan Kla* pan lysine lactylation

To confirm that FASN was indeed hyperlactylated when the level of lactate was upregulated, we treated differentiated 3T3-L1 cells with different concentrations of lactate. Murine 3T3-L1 preadipocytes were differentiated into mature adipocytes during a 10-day culture period, and then, the cells were treated with 0 mM, 3 mM, or 10 mM sodium lactate for 24 h (Fig. 4A). Lactate treatment significantly increased the protein level of FASN while FASN mRNA had no statistical significance (Fig. 4B, C). Western blot analysis of lactyllysine demonstrated that the lactylation level was significantly upregulated in cells with increasing lactate concentration (Fig. 4D). We immunoprecipitated FASN from treated 3T3-L1 cells and analyzed the lactylation level via western blotting using an anti-lactyllysine antibody. FASN was highly lactylated in cells treated with 10 mM lactate (Fig. 4E). These results show that lactate treatment upregulated FASN expression and that the lactylation level of FASN was significantly increased.

### Lactylation inhibites the activity and function of FASN

**Fig. 5:**
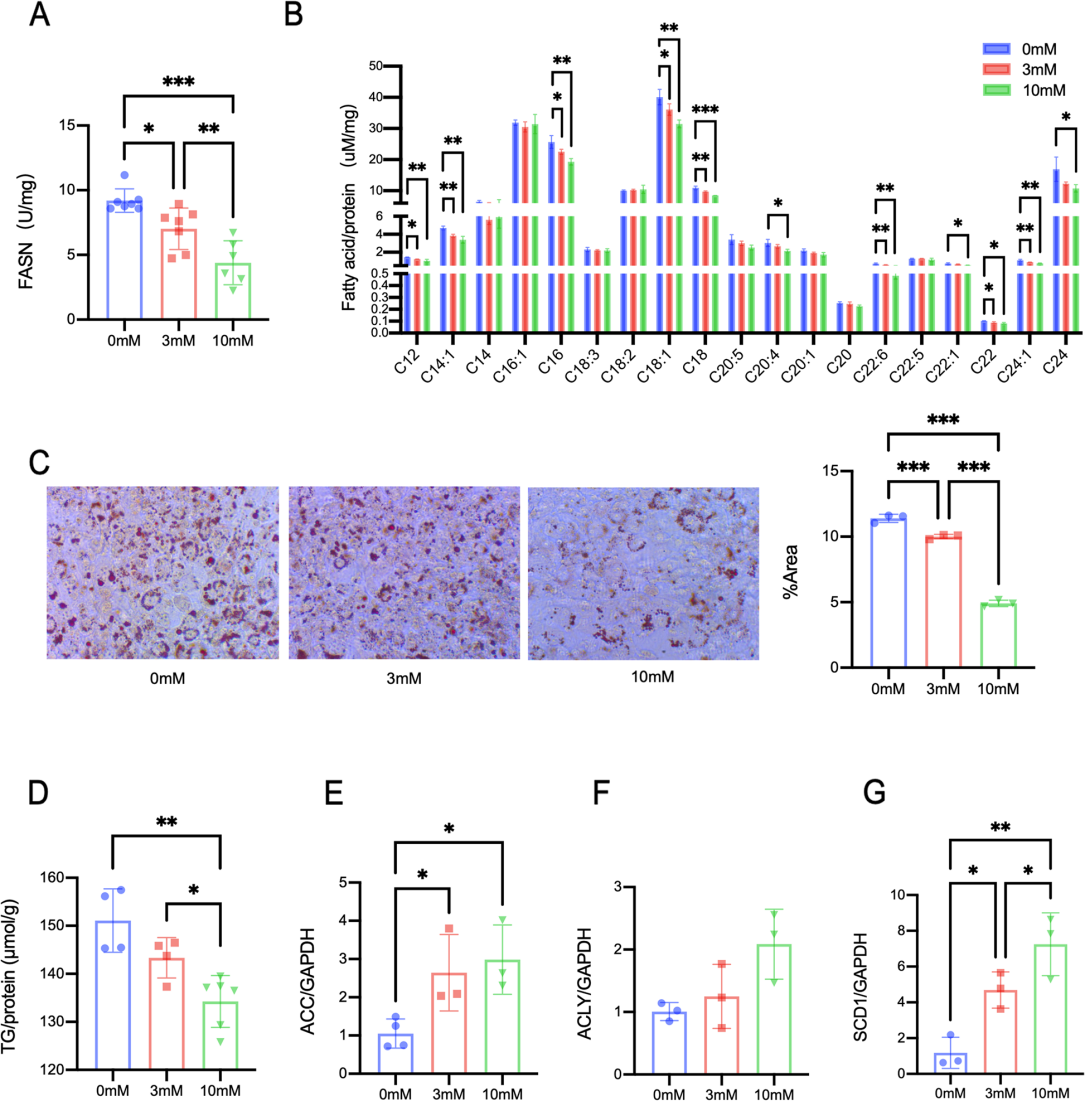
Lactate treatment inhibites FASN activity and lipid synthesis in 3T3-L1 cells. **A** The change in FASN activity in 3T3-L1 cells. **B** Profile of fatty acids in 3T3-L1 cells measured by UPLC–MS. **C** Intracellular lipid droplets were stained with Oil Red O and visualized via microscopy at 40 × magnification. **D** Triglyceride levels in 3T3-L1 cells. **E–H** Marker gene of de novo lipogenesis. Data are shown as mean ± SD, *n* = 3–4. *p < 0.05, **p < 0.01; ***p < 0.001. GAPDH glyceraldehyde-3-phosphate dehydrogenase, FASN fatty acid synthase, SCD1 stearoyl-CoA desaturase 1, ACLY ATP citrate lyase, ACC acetyl-CoA carboxylase, TG triglyceride

We wanted to determine the consequence of increased FASN lactylation levels. In 3T3-L1 cells, FASN activity was repressed with increasing lactate concentration (Fig. 5A). Long-chain fatty acids and medium-chain fatty acids were decreased. Palmitate, a C16:0 fatty acid is a specific product of FASN, and its concentration was significantly reduced after treatment (Fig. 5B). The results of oil red O staining showed that lipid accumulation in cells decreased with increasing lactate concentration (Fig. 5C). The triglyceride level showed the same trend (Fig. 5D). These findings indicated that lactate inhibited lipid synthesis in cells. Next, we determined the expression of enzymes related to de novo lipid synthesis. The mRNA expressions of acetyl-CoA carboxylase (ACC) and stearoyl-CoA desaturase 1 (SCD1) were upregulated in the 3 mM and 10 mM groups, while ACLY mRNA showed no different (Fig. 5E–G). These results show that the regulatory effect of lactate on enzymes is complex. On the one hand, it can promote the expression of enzymes, but at the same time, it inhibits FASN function through posttranslational modification and decreases de novo lipid synthesis.

## Discussions

In the present study, we observed that exercise-induced lactate was essential for fat–mass loss when using the lactate inhibitor DCA. Our data showed that in the HIIT mice, protein lactylation levels were increased in multiple tissues; in contrast, the level of protein lactylation was reduced after the lactate production inhibitor DCA was administered. Among the changes, the alterations in subcutaneous adipose tissue were very significant, suggesting that HIIT-induced lactylation is involved in the metabolic regulation of adipose tissue. After comprehensive proteome analyses and quantitative proteomics analyses of lysine lactylation in subcutaneous adipose tissue, FASN was identified as the major target protein regulated by lactylation. In vitro stimulation of 3T3-L1 cells with lactate treatment also increased the lactylation levels of FASN, repressed FASN activity, while the levels of palmitate and triglyceride decreased, which are downstream products of FASN regulation, suggesting that FASN function was inhibited by lactylation. The higher levels of lactate produced by HIIT induce FASN lactylation in adipose tissue and inhibit de novo lipid synthesis, which may be a key mechanism by which HIIT contributes to fat mass loss.

Compared with the classic moderate-intensity, long-term fat loss exercise protocol, HIIT uses glycogen and glucose as energy sources and the glycolytic pathway as the major energy supply mode. The ratios of respiratory gas exchange at rest and during exercise indicate that HIIT does not consume fat [10, 11]; in addition, HIIT has a short exercise duration and low total lipid consumption [12, 13]. How does it achieve a fat–mass loss effect? Some studies have reported that HIIT can significantly increase postexercise oxygen consumption [14]. The impact of exercise on lipid metabolism and glucoregulation persists after exercise [15–17]. Interval-walking results in higher EPOC, free fatty acids, glycerol concentrations, and glycerol kinetics during and after exercise in T2D subjects [18]. These findings strongly suggest that modulation of lipid metabolism by HIIT occurs after exercise.

The mechanism of HIIT regulating lipid metabolism is less known. Studies have found that after the intervention of HIIT, the fat mass of elderly rats is significantly decreased, and the expressions of HSL and ATGL in adipose tissue are enhanced [19]. A large amount of glucose was used to supply energy during HIIT, which decreases blood glucose levels. DNL in adipose tissue was inhibited to cope with the body’s large demand for energy supply [20]. In this study, HIIT increased FA uptake and potentially reduced lipolysis that suggested HIIT might influence lipid metabolism through a complicated system.

Lactate, as a myokine and exerkine, plays an important biological role through diverse molecular mechanisms [21]. Many studies have shown that exercise-induced improvement in glucose and lipid metabolism and reduction in body fat in mice is dependent on skeletal muscle lactate production, and injection of the lactate inhibitor DCA leads to disappearance of the exercise-induced improvements [22]. Lactate and its receptor GPR81 decrease cAMP levels in adipocyte to inhibit lipolysis in an autocrine and paracrine loop [23]. Exercise could induce the biosynthesis of Lac-Phe from lactate and phenylalanine to control dietary intake and regulate the balance of energy and metabolism [24]. Furthermore, rats treated with exercise training and the compounds lactate and caffeine showed decreased epididymal and scapular fat mass compared to sedentary rats, suggesting that administration of lactate can effectively decrease fat mass [25]. In this study, after inhibiting elevation in blood lactate levels, the fat-reducing effect induced by HIIT was attenuated, indicating that lactate plays an important role in the fat-reducing effect of HIIT.

In vivo, concentration and treatment time of lactate will make different results in lipid metabolism. Lactate predominantly stimulated differentiation and triglyceride levels during the early stages of differentiation (days 0–2) in 3T3-L1 cells [26]. Three hours treatment of lactate inhibited glycerol and fatty acid release in differentiated 3T3-L1 cells [27]. In our study, lactate decreased lipogenesis in 3T3-L1 cells after 24 h treatment, indicating that different concentrations and incubation can change the function of lactate.

Lactylation is a novel identified type of acylation, and elevated lactate levels induces protein lactylation in the body [28]. It exists widely in different organisms and occurs in the nucleus, cytoplasm, and mitochondria [29]. Histone lysine lactylation has been studied extensively, and its regulation has been the primary focus to date. In contrast to the histone lysine lactylation in the nucleus, much less is known about nonhistone lysine lactylation. HIIT repeatedly exposes the internal milieu to a high lactate environment; does it induce protein lactylation? Our study found for the first time that HIIT can induce an enhancement in protein lactylation, while there is a certain specificity. Lactylation was significantly enhanced in iWAT, liver, and heart, but no significant lactylation enhancement was found in visceral fat or soleus and gastrocnemius muscles, and the tissue specificity of its effects is unclear. In addition to the regulation of lactylation by lactate levels, acylase/deacylase activity also plays an important role. There are many types of acyltransferases, of which adenoviral E1A binding protein of 300 kDa (P300) is the most characteristic and the most powerful broad-spectrum lysine acyltransferase. Some studies have reported that lactylation is a type of p300-dependent acylation [30]. A previous study confirmed that class I histone deacetylases (HDAC1–3) are responsible for the reversible and dynamic regulation of histone lactylation, that is, HDAC1–3 are histone lactylation delactylases [31]. The distribution of HDAC1-3 in tissues, the regulation of nonhistone lactylation, and whether exercise has tissue-specific regulatory effects on these parameters require more research to confirm.

Moreover, the relationship between hyperlactylation and lipid metabolism in iWAT remains unclear. To understand the mechanism underlying the function of lactylation and lipid metabolism, we performed a quantitative lactylation proteomics analysis. We identified 13 proteins with increased lysine lactylation in HIIT mice and decreased lysine lactylation in DCA + HIIT mice using quantitative proteomics. Among the identified proteins, FASN was found to have the most lactylation sites (at 4 Lys residues). In 3T3-L1 adipocytes, FASN was highly lactylated in the presence of lactate, suggesting that FASN lactylation plays a key role in regulating lipid metabolism.

Circulating triglyceride and de novo lipogenesis (DNL) are two main sources of fatty acids in adipose tissues. Through DNL, carbohydrates are converted to fatty acids. FASN, the important rate-limiting enzyme in DNL, is a complex multifunctional enzyme that can convert acetyl-CoA and malonyl-CoA into palmitate. The activity and protein stability of FASN are regulated by posttranslational modifications, including ubiquitination, sumoylation, and acetylation. Phosphorylation inhibits FASN activity. When intracellular energy depletion, nutrient deprivation, or hypoxia occurs, AMPK phosphorylates FASN in adipocytes, which results in less palmitate production. FASN is directly modified by O-GlcNAcylation in the liver. O-GlcNAcylation of FASN increases the interaction between FASN and ubiquitin specific protease-2A (USP2A), leading to inhibition of FASN ubiquitination and enhancement of FASN stability [32]. This leads to upregulation of FASN levels and enhancement of DNL in the liver. The acetylation of FASN is controlled by lysine acetyltransferase 8 (KAT8) and deacetylase 3. FASN acetylation induces FASN interaction with the E3 ubiquitin ligase tripartite motif containing 21 (TRIM21) and reduces the stability of FASN, which results in decreased DNL [33]. The effect of lactylated FASN on its function has not been reported to date.

In the present study, we found that lactate treatment significantly upregulated the expression and protein level of FASN, and the expression levels of enzymes that regulate DNL were also significantly increased, but FASN activity, triglycerides, and palmitate (a specific product of FASN) were significantly reduced in cells. These findings suggest that lactylation, as a negative feedback regulation mechanism, inhibits FASN activity and DNL. In addition to transcriptional regulation and posttranscriptional control, PTMs are another powerful regulatory method and ultimately determine the activity of an enzyme. The present work represents a significant expansion of our current understanding of lactylation and demonstrates that lactylation plays a key role in connecting glucose metabolism and lipid metabolism.

It is a limitation that we did not add the DCA group in the experimental design. It has been shown that DCA injection does not affect the exercise ability and weight of mice, and only inhibits the increase of lactate concentration during exercise training [22]. Reduction of lactate is one of the effects of DCA. DCA treatment could inhibit the activity of pyruvate dehydrogenase kinase which result in changes of a wide range of metabolites. Therefore, we might consider specific inhibitors to inhibit lactate dehydrogenase activity or using genetic mice models for further researches.

## Conclusions

In summary, our study identified protein lactylation is probably one factor that controls fat loss in exercise. Combining mass spectrometry analysis and bioinformatics tools, we found that FASN, an important enzyme in DNL, participates in HIIT-induced fat loss. Furthermore, we showed that FASN lactylation reduces its activity. Beyond that whether lactylation is regulated by exercise in a tissue-specific manner, the activity of protein lactylation in other tissues and the effect of lactylation on metabolic diseases are currently unclear and merit investigation in the future.

## Methods

### Animals and exercise protocol

Fifty-four 8-week-old male C57B/L6 mice (specific pathogen-free level) were provided by Beijing Vital River Laboratory Animal Technology Co. (Beijing, China). Animal studies were performed in accordance with the Guiding Principles for Research Involving Animals and Human Beings [34]. During the experiment, mice had free access to water and food. Animals were housed in pairs on a 12-h/12-h light–dark cycle in a temperature-controlled room at 22 ± 2 °C.

We randomly divided mice into 3 groups: (1) control group (*n* = 18), no exercise; (2) HIIT group (*n* = 18), HIIT; and (3) DCA + HIIT group (*n* = 18), DCA (dichloroacetate) injection and HIIT. Exercise intervention was performed 5 days per week for 8 weeks. Mice were trained on a treadmill. The mice ran at 15 m/min for 30 min in the first adaptation week. An exercise capacity test was performed before the formal training protocol to determine the maximal running speed. Mice began running at 8 m/min and speed was increased by 2 m/min every 2 min until the mice could not keep up with the speed for more than 10 s [35]. The speed was recorded. The HIIT group began treadmill running at 40% Vmax for 5 min, then ran at 85% Vmax for 1.5 min followed by 2 min of rest at 45% Vmax 9 times and finished with a 5-min treadmill running at 40% Vmax [36]. DCA (400 mg/kg, intraperitoneal injection, 08168-10EA, Sigma–Aldrich, Darmstadt, Germany), an inhibitor of lactate production, was injected intraperitoneally 10 min before HIIT [22]. None of the mice died during HIIT.

Body weight was measured, and food intake was recorded; 24 h after the last exercise, mice were fasted overnight but had free access to water and were anesthetized with pentobarbital sodium (100 mg/kg, intraperitoneal injection, 57–33-0, Sigma–Aldrich, Darmstadt, Germany). Blood samples were drawn from the heart. The soleus and gastrocnemius muscle, heart, liver, inguinal white adipose tissue (iWAT), epididymal white adipose tissue (eWAT), and brown adipose tissue (BAT) were excised and weighed. iWAT was removed from the inguinal region, eWAT from the epididymal fat deposits, and BAT from the shoulder blades. All tissue samples were stored at − 80 °C.

### Fatty acid determination

Fatty acids were detected via ultra-performance liquid chromatography (UPLC). Cell and plasma solutions were placed into 1.5 mL tubes, and 150 µL of cold CH_3_CN was added for protein precipitation. Then, the solutions were vortexed and centrifuged at 15,000 × *g* for 10 min at 4 °C. The supernatants were transferred to clean tubes and evaporated. Sample analysis was carried out on an Acquity-Xevo TQS (Waters, Milford, USA) system.

### Histological staining

The tissues were fixed in 4% paraformaldehyde for 48 h and then embedded in paraffin, cut into 4-µm-thick sections and stained with H&E (ZLI-9610, Zhongshan Golden Bridge Biotechnology, Beijing, China). Finally, the tissue sections were viewed under a ZEISS ImagerM1 microscope (Carl Zeiss Jena GmbH, Planetariums, Jena, Germany). The cross-sectional area of adipocytes was measured in > 10 randomly selected fields, and the average area was calculated.

### Cell culture and treatment

DMEM (SH30022.01, Hyclone, UT, USA) supplemented with 10% newborn calf serum (NCS, 16010159, Gibco, CA, USA) and penicillin (100 U/ml)-streptomycin solution (0.1 mg/ml, P1400, Solarbio, Beijing, China) was used to culture 3T3-L1 cells (1101MOU-PUMC000155, BMCR, Beijing, China) at 37 °C in a humidified atmosphere of 5% CO_2_. After reaching sufficient confluence, the cells were treated with DMEM containing 10% NCS, 2 µg·mL^−1^ insulin (I5500, Sigma–Aldrich, Darmstadt, Germany), 1 µM dexamethasone (D1756, Sigma–Aldrich, Darmstadt, Germany), 0.5 mM troglitazone (HY-50935, MCE, NJ, USA), and 0.5 mM IBMX (I7018, Sigma–Aldrich, Darmstadt, Germany) for 2 days. Then, the medium was replaced with maturation medium (DMEM, 10% NCS, 2 µg·mL^−1^ insulin and 0.5 mM troglitazone) for 8 days. On the 10th day, the cells were treated with different concentrations of sodium lactate (1614308, Sigma–Aldrich, Darmstadt, Germany) for 24 h. Concentrations of cell triglycerides were determined using commercially available assay kits (E1025-105, Applygen Technologies Inc., Beijing, China).

### Oil red O staining

On Day 11, 3T3-L1 cells were washed twice with PBS and fixed for 2 h with 4% formaldehyde at 4 °C. After two washes with PBS, the cells were stained with 1.5% oil red O (G1262-4, Solarbio, Beijing, China) for 1 h at 60 °C. Thereafter, the cells were washed with ddH_2_O twice and photographed with a ZEISS inverted fluorescence microscope (Carl Zeiss Jena GmbH, Planetariums, Jena, Germany).

### Determination of fatty acid synthase (FASN) activity

FASN activity was determined with a Fatty Acid Synthetase Activity Assay Kit (BC0555, Solarbio, Beijing, China). Briefly, 3T3-L1 cells were disrupted by sonication for 5 min in an ice bath and then centrifuged at 12,000 × *g* for 20 min at 4 °C. The supernatant was transferred to a tube, and FASN activity was evaluated according to the assay kit instructions [37].

### Quantitative real-time PCR

Total RNA was isolated and reverse-transcribed using a reverse transcription system (DP419, TianGen Biotech, Beijing, China). After treatment, total RNA was extracted from 3T3-L1 cells using TRIzol (R1100, Solarbio, Beijing, China) and reverse transcribed using a FastQuant RT Kit (KR106, TianGen Biotech, Beijing, China). All amplification reactions involved were performed with TransStart® Green qPCR SuperMix (FP205, TransGen Biotech, Beijing, China) in a LightCycler 96 Quantitative PCR System (LightCycler 96, Roche, Basle, Switzerland). After denaturation at 95 °C for 30 s, the solution was subjected to PCR at 95 °C for 5 s, 60 °C for 15 s, and 72 °C for 10 s for 45 cycles. Glyceraldehyde-3-phosphate dehydrogenase (GAPDH) was used as an internal control. The primers are listed in Supplemental Table 1. The results were analyzed with the delta-delta Ct method.

### Western blotting

The iWAT, eWAT, liver, heart, and soleus and gastrocnemius muscles were collected from C57B/L6 mice. 3T3-L1 cells were collected after treatment. Cells and tissues were homogenized in lysis buffer. Total protein was quantified by Pierce™ BCA Protein Assay Kit (23227, Thermo Scientific, MA, USA). Protein solutions were separated via electrophoresis using 10–12% Bis–Tris gels and transferred to polyvinylidene difluoride (PVDF, 88518, Thermo Scientific, MA, USA) membranes. The membranes were incubated with 5% skim milk for 1 h and then incubated overnight at 4 °C with primary antibody (FASN rabbit mAb, 1:1000, C20G5, Cell Signaling Technology, Ltd., Boston, USA; anti-L-lactyl lysine rabbit mAb, 1:1000, PTM-1401, Jingjie PTM BioLab Co. Ltd., Hangzhou, China; GAPDH, 1:8000, CST5174, Cell Signaling Technology, Boston, USA) diluted in TBST and finally incubated for 1 h with secondary antibody (goat anti-rabbit IgG, 1:8000, E030110, Earthox, CA, USA) diluted with TBST. Chemiluminescence reagent (WBKLS0100, Millipore, Burlington, USA) and a luminescence imaging analyzer (Amersham Imager 680, General Electric, Boston, USA) were used for protein band detection. The relative protein expression was analyzed based on the gray value for GAPDH.

### Immunoprecipitation assays

Immunoprecipitation was measured using a co-immunoprecipitation Kit (abs955, Absin, Beijing, China) according to the manufacturer’s instructions. After treatment, cells were collected and lysed at 4 °C for 10 min. The supernatant was centrifuged and quantified using a BCA kit. Then, 500 µg of total cellular proteins were incubated with FASN rabbit mAb (1:50) and rotated overnight at 4 °C; 10 µL of Protein A/G-agarose beads was added, and the mixture was shaken for 3 h at 4 °C. The precipitates were washed 3 times by wash buffer and boiled in SDS buffer. The proteins of supernatant were separated via SDS–PAGE and detected with anti-L-lactyl lysine rabbit mAb antibody.

### 4D label-free lactylation quantitative proteomics and analysis

4D label-free lactylation quantitative proteomics in mouse iWAT was performed by Jingjie PTM BioLab Co., Ltd. (Hangzhou, China).

#### Protein extraction and trypsin digestion

Samples were removed from − 80 °C and then fully ground to powder with liquid nitrogen. The samples in each group were added to 4 times the volume of extraction buffer (containing 10 mM DL-dithiothreitol, 1% protease inhibitor cocktail, 3 μM TSA, 50 mM NAM) for ultrasonic lysis. An equal volume of Tris balanced phenol was added, and the samples were centrifuged at 5500* g* at 4 °C for 10 min. The supernatant was taken, and 5 times the volume of 0.1 M ammonium acetate/methanol was added for precipitation overnight. The precipitate was washed successively with methanol followed by acetone. Finally, 8 M urea was used for redissolution, and the protein concentration was determined using a BCA kit.

The protein in each sample was enzymatically hydrolyzed in equal quantities, and the volume was adjusted to be consistent with the lysate. Then, 20% TCA was slowly added, and the sample was vortexed and allowed to precipitate at 4 °C for 2 h. Centrifugation was performed at 4500 × *g* for 5 min, and the precipitate was washed with precooled acetone 2–3 times. After drying off the precipitate, 200 mM TEAB was added. Then, trypsin was added at a ratio of 1:50 (protease:protein, M/M), and the sample was digested overnight. Dithiothreitol (DTT) was added to a final concentration of 5 mM, and the sample was reduced at 56 °C for 30 min. Then, iodine acetamide (IAA) was added to a final concentration of 11 mM, and the mixture was incubated for 15 min at room temperature away from light.

#### Panlactylation antibody-based PTM enrichment

Peptides were dissolved in IP buffer solution (100 mM NaCl, 1 mM EDTA, 50 mM Tris–HCl, 0.5% NP-40, pH 8.0), and the supernatant was transferred to prewashed lactylation resin (antibody resin Art. 5798717296493442124, from Hangzhou Jingjie Biotechnology Co., Ltd., PTM Bio), placed on a rotating shaker at 4 °C, and gently shaken overnight. After incubation, the resin was washed 4 times with IP buffer solution and twice with deionized water. Finally, 0.1% trifluoroacetic acid was used to elute the resin-bound peptide three times. The eluent was collected and vacuum dried. After being drained, the salt was removed according to the C18 ZipTips instructions (ZTC18S, Millipore, Burlington, USA), and after being vacuum-frozen and drained, the liquid was obtained for mass analysis.

#### Quantitative proteomic analysis via LC–MS/MS

The peptides were dissolved in buffer A and separated via ultra-performance liquid chromatography using a nanoElute system. Buffer A was 0.1% formic acid and 2% acetonitrile in water. Buffer B was 0.1% formic acid in acetonitrile solution. The liquid phase gradient settings were as follows: 0–44 min, 6–22% B; 44–56 min, 22–30% B; 56–58 min, 30–80% B; 58–60 min, 80% B. The flow rate was maintained at 300 nL/min. The peptides were separated and then injected into the capillary ion source and analyzed using a timsTOF Pro mass spectrometer.

#### Bioinformatics analysis

Gene Ontology (GO) annotation of the proteome was performed with eggnog-mapper software (version 2.0). Identified protein domain functional descriptions were annotated using InterProScan (version 5.14–53.0) based on the protein sequence alignment method, and the InterPro domain database was used. The Kyoto Encyclopedia of Genes and Genomes (KEGG) database was used to annotate protein pathways. These pathways were classified into hierarchical categories according to the KEGG website. All differentially expressed protein database accessions or sequences were searched against the STRING database (version 11.0) for protein–protein interactions. Wolfpsort (version 0.2), a subcellular localization prediction software, was used to predict subcellular localization. Soft MoMo (version 5.0.2) was used to analyze the model of sequences comprising amino acids in specific positions of modify-21-mers in all protein sequences.

### Quantification and statistical analysis

Data are presented as the mean ± SD in this study. Statistical analyses were performed by one-way ANOVA followed by the Newman–Keuls posttest. A *p* value < 0.05 was considered statistically significant. GraphPad Prism (Version 9.0; GraphPad Software, La Jolla, CA, USA) was used to verify the normality of the data. The cross-sectional area of adipocytes was calculated by ImageJ (Version 1.53a.). The grayscale values of the protein bands were calculated by image processing and analysis in Image Processing and Analysis in Java (ImageJ, version 1.53a).

### Supplementary Information

**Additional file 1: Fig. S1.** HIIT and DCA injection change the plasma fatty acid content and lipid metabolism in iWAT. **Fig. S2.** HIIT promotes protein lactylation in different tissues. **Fig. S3.** Supplement detection and profile of lysine lactylation in mice iWAT.

**Additional file 2: Supplemental Table 1.** List of primers used for the PCR analysis.

## Data Availability

The mass spectrometry proteomics data have been deposited to the ProteomeXchange Consortium via the iProX partner repository with the dataset identifier PXD044919 (https://www.iprox.cn/page/project.html?id=IPX0006989000).

## Abbreviations

2PG: 2-Phospho-d-glycerate
ACC: Acetyl-CoA carboxylase
ACLY: ATP citrate lyase
ATGL: Adipose triglyceride lipase
BAT: Brown adipose tissue
CD36: Cluster of differentiation 36
CPT-1: Carnitine palmitoyltransferase-1
DCA: Dichloroacetate
DCA + HIIT: Dichloroacetate injection + high-intensity interval training
DNL: De novo lipogenesis
ENO: Enolase
eWAT: Epididymal white adipose tissue
FABP3: Fatty acid binding protein 3
FASN: Fatty acid synthase
GAPDH: Glyceraldehyde-3-phosphate dehydrogenase
GPR81: Gi-protein-coupled receptor 81
HDAC1–3: Class I histone deacetylases
HIIT: High-intensity interval training
HSL: Hormone-sensitive lipase
iWAT: Inguinal white adipose tissue
KAT8: Lysine acetyltransferase 8
KEGG: Kyoto Encyclopedia of Genes and Genomes
MCT-1: Monocarboxylate transporter-1
P300: E1A binding protein of 300 kDa
Pan Kla: Pan lysine lactylation
PVDF: Polyvinylidene difluoride
SCD1: Stearoyl-CoA desaturase 1
TG: Triglyceride
TRIM21: Tripartite motif containing 21
UPLC: Ultra-performance liquid chromatography
USP2A: Ubiquitin specific protease-2A
