# Supplementary material for: High-intensity interval training induces lactylation of fatty acid synthase to inhibit lipid synthesis
Source: BMC Biol. 2023 Sep 19;21:196. doi: 10.1186/s12915-023-01698-9 (PMC10510295; doi:10.1186/s12915-023-01698-9)
Supplement: Supplementary file 1 — Additional file 1: Fig. S1. HIIT and DCA injection change the plasma fatty acid content and lipid metabolism in iWAT. Fig. S2. HIIT promotes protein lactylation in different tissues. Fig. S3. Supplement detection and profile of lysine lactylation in mice iWAT. [file 12915_2023_1698_MOESM1_ESM.docx]

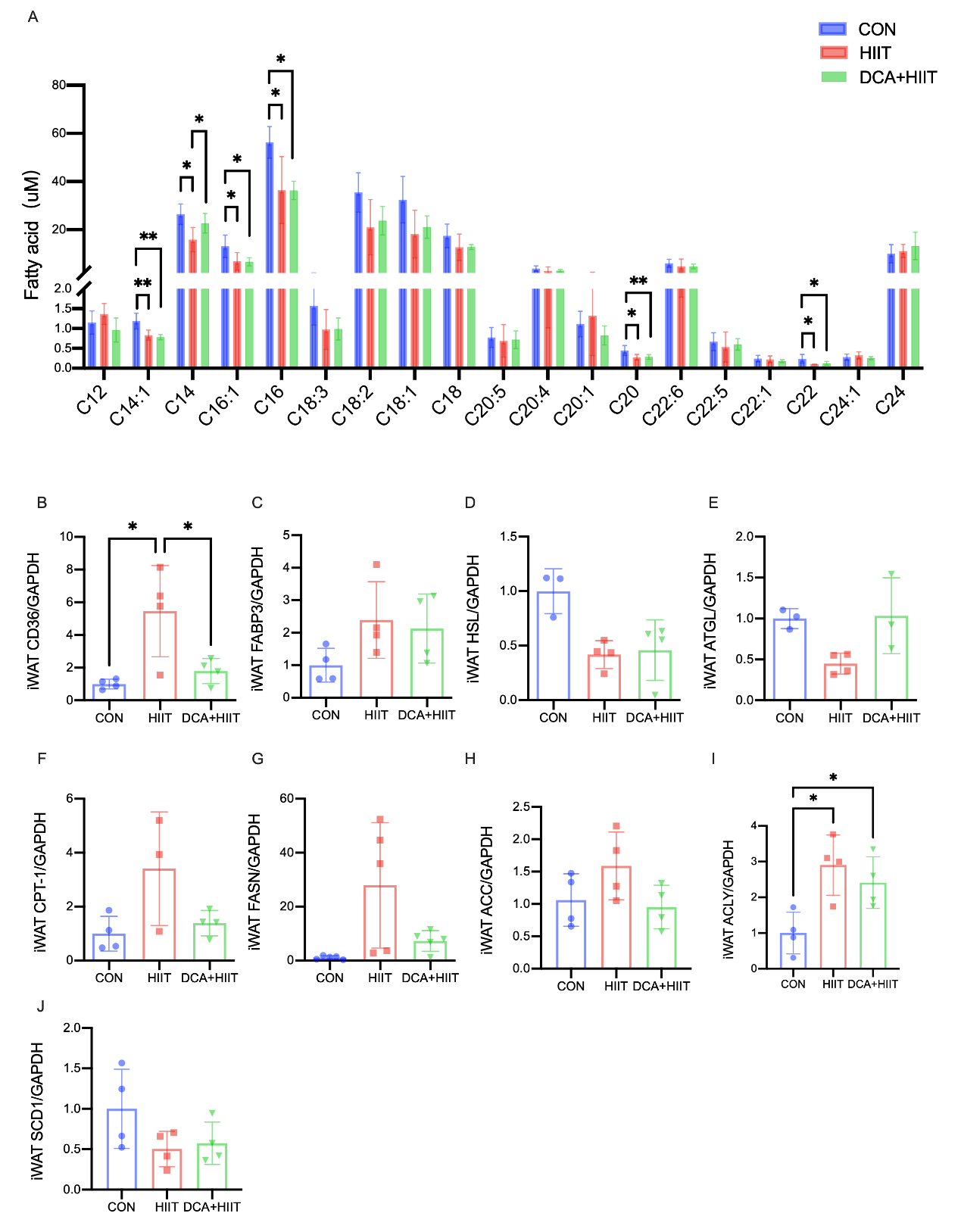


**Fig. S1 HIIT and DCA injection change the plasma fatty acid content and lipid metabolism in iWAT.**


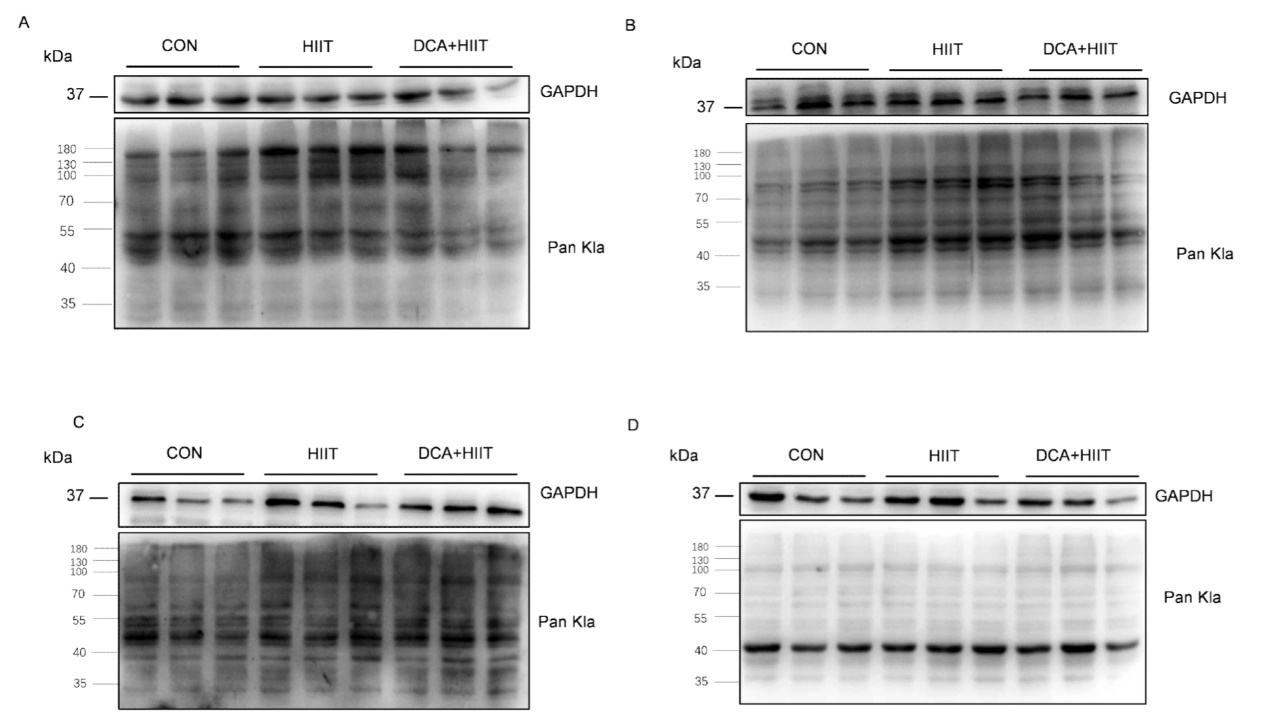


**Fig. S2** **HIIT promotes protein lactylation in different tissues.**


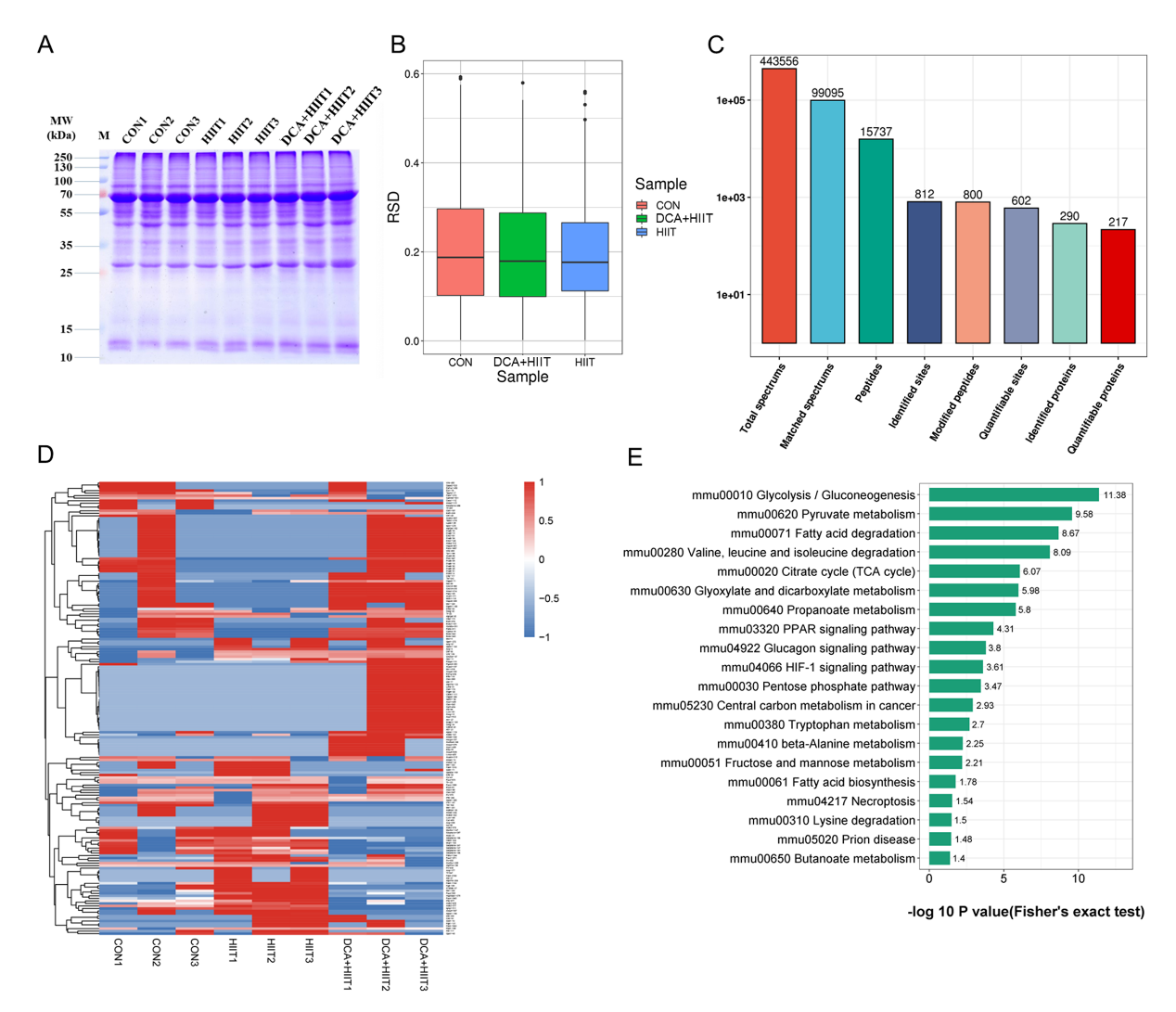


**Fig. S3 Supplement detection and profile of lysine lactylation in mice iWAT.**
