# Supplementary material for: High-intensity interval training induces lactylation of fatty acid synthase to inhibit lipid synthesis
Source: BMC Biol. 2023 Sep 19;21:196. doi: 10.1186/s12915-023-01698-9 (PMC10510295; doi:10.1186/s12915-023-01698-9)
Supplement: Supplementary file 2 — Additional file 2: Supplemental Table 1. List of primers used for the PCR analysis. [file 12915_2023_1698_MOESM2_ESM.docx]

**Supplemental Table 1**

**List of primers used for the PCR analysis.**

| Gene | Sequence (5’-3’) |
| --- | --- |
| GAPDH | Forward 5’- ACA GCA ACA GGG TGG TGG AC-3’ |
|  | Reverse 5’- TTT GAG GGT GCA GCG AAC TT-3’ |
| FASN | Forward 5’- ACC TCT CCC AGG TGT GTG AC -3’ |
|  | Reverse 5’- AGT TCC TGC ACT CAG GGT GT -3’ |
| HSL | Forward 5’- GGA GTC TAT GCG CAG GAG TG -3’ |
|  | Reverse 5’- GCT TCT TCA AGG TAT CTG TGC C -3’ |
| ATGL | Forward 5’- TGT GGC CTC ATT CCT CCT AC-3’ |
|  | Reverse 5’- TCGTGGATGTTGGTGGAGCT-3’ |
| SCD1 | Forward 5’-GGCTAGCTATCTCTGCGCTC-3’ |
|  | Reverse 5’-GAACTGCGCTTGGAAACCTG-3’ |
| ACLY | Forward 5’-TTCGTCAAACAGCACTTCC-3’ |
|  | Reverse 5’-ATTTGGCTTCTTGGAGGTG-3’ |
| ACC | Forward 5’-CAGTAACCTGGTGAAGCTGGA-3’ |
|  | Reverse 5’-GCCAGACATGCTGGATCTCAT-3’ |
| CD36 | Forward 5’- AGG TCT ATC TAC GCT GTG TTC G -3’ |
|  | Reverse 5’- TGG TTG TCT GGA TTC TGG AG -3’ |
| FABP3 | Forward 5’- TTC AGC TGG GAA TAG AGT TCG -3’ |
|  | Reverse 5’- CTG CAC ATG GAT GAG TTT GC -3’ |
| CPT-1 | Forward 5’- GGC ACC TCT TCT GCC TTT AC-3’ |
|  | Reverse 5’-TTT GGG TCA AAC ATG CAG AT-3’ |

Abbreviations: GAPDH=glyceraldehyde-3-phosphate dehydrogenase, FASN=fatty acid synthase, HSL=hormone-sensitive lipase, ATGL=adipose triglyceride lipase, SCD1=stearoyl-CoA desaturase 1, ACLY=ATP citrate lyase, ACC=acetyl-CoA carboxylase, CD36=cluster of differentiation 36, FABP3=fatty acid binding protein 3, CPT-1=carnitine palmitoyltransferase-1.
